# Supplementary material for: Real time, in vivo measurement of neuronal and peripheral clocks in Drosophila melanogaster
Source: eLife. 2022 Oct 3;11:e77029. doi: 10.7554/eLife.77029 (PMC9662830; doi:10.7554/eLife.77029)
Supplement: Supplementary file 1. — For LABL experiments, stocks are maintained with one parental line expressing both the Gal4 driver and LABL reporter, and the other parental line expressing UAS-FLP. [file elife-77029-supp1.docx]

**Supplementary File 1.** Genotypes of flies used in the figures. For LABL experiemnts, stocks are maintained with one parental line expressing both the Gal4 driver and LABL reporter, and the other parental line expressing UAS-*FLP*.

| **Figure** | **Label** | **Genotype** |
| --- | --- | --- |
| Figure 2 | tim-UAS-Gal4 | w ; tim-UAS-Gal4 / + ; LABL / UAS-FLP2 |
| Figure 3A | tim-UAS-Gal4 | w ; tim-UAS-Gal4 / + ; LABL / UAS-FLP2 |
| Figure 3A | plo | w ; plo ; + |
| Figure 3A | PER-BG::Luc | w ; per-BG-luc ; + |
| Figure 3B | *tim^01^* | w ; tim-UAS-Gal4, *tim^01^* / *tim^01^* ; LABL / UAS-FLP2 |
| Figure 3C | *han^5304^* | w,*han^5304^* ; tim-UAS-Gal4 / + ; LABL / UAS-FLP2 |
| Figure 4A, 4B | tim-UAS-Gal4 | w ; tim-UAS-Gal4 / + ; LABL / UAS-FLP2 |
| Figure 4A, 4B | Pdf-Gal4 | w ; Pdf-Gal4 / + ; LABL / UAS-FLP2 |
| Figure 4A, 4B | DvPdf-Gal4 | w ; DvPdf-Gal4 / + ; LABL / UAS-FLP2 |
| Figure 4A, 4B | R18H11-Gal4 | w ; LABL / + ; R18H11-Gal4 / UAS-FLP2 |
| Figure 6B, 6C | tim-UAS-Gal4 | w ; tim-UAS-Gal4 / + ; LABL / UAS-FLP2 |
| Figure 6B, 6C | Pdf-Gal4 | w ; Pdf-Gal4 / + ; LABL / UAS-FLP2 |
| Figure 6B, 6C | DvPdf-Gal4 | w ; DvPdf-Gal4 / + ; LABL / UAS-FLP2 |
| Figure 6B, 6C | R18H11-Gal4 | w ; LABL / + ; R18H11-Gal4 / UAS-FLP2 |
| Figure 6B | Clk4.1-Gal4 | w ; LABL / + ; Clk4.1-Gal4 / UAS-FLP2 |
| Figure 6B | Mai179-Gal4 | w ; Mai179-Gal4 / + ; LABL / UAS-FLP2 |
| Figure 6B | Clk9M-Gal4 | w ; Clk9M-Gal4 / + ; LABL / UAS-FLP2 |
| Figure 6D | Pdf-Gal4 | w ; Pdf-Gal4 / + ; + / G-TRACE |
| Figure 6D | DvPdf-Gal4 | w ; DvPdf-Gal4 / + ; + / G-TRACE |
| Figure 6D | R18H11-Gal4 | w ; + / + ; R18H11 / G-TRACE |
| Figure 7A-7D | Pdf-Gal4 | w ; Pdf-Gal4 / + ; LABL / UAS-FLP2 |
| Figure 7A-7D | DvPdf-Gal4 | w ; DvPdf-Gal4 / + ; LABL / UAS-FLP2 |
| Figure 7A-7D | R18H11-Gal4 | w ; LABL / + ; R18H11-Gal4 / UAS-FLP2 |
| Figure 7A-7D | Clk4.1-Gal4 | w ; LABL / + ; Clk4.1-Gal4 / UAS-FLP2 |
| Figure 7A-7D | Mai179-Gal4 | w ; Mai179-Gal4 / + ; LABL / UAS-FLP2 |
| Figure 7A-7D | Clk9M-Gal4 | w ; Clk9M-Gal4 / + ; LABL / UAS-FLP2 |
| Figure 7A-7D | Pdf-Gal4 \| *han^5304^* | *han^5304^* ; Pdf-Gal4 / + ; LABL / UAS-FLP2 |
| Figure 7A-7D | DvPdf-Gal4 \| *han^5304^* | *han^5304^* ; DvPdf-Gal4 / + ; LABL / UAS-FLP2 |
| Figure 7A-7D | R18H11-Gal4 \| *han^5304^* | *han^5304^* ; LABL / + ; R18H11-Gal4 / UAS-FLP2 |
| Figure 7A-7D | Clk4.1-Gal4 \| *han^5304^* | *han^5304^* ; LABL / + ; Clk4.1-Gal4 / UAS-FLP2 |
| Figure 7A-7D | Mai179-Gal4 \| *han^5304^* | *han^5304^* ; Mai179-Gal4 / + ; LABL / UAS-FLP2 |
| Figure 7A-7D | Clk9M-Gal4 \| *han^5304^* | *han^5304^* ; Clk9M-Gal4 / + ; LABL / UAS-FLP2 |
| Figure 7B-7C | tim-UAS-Gal4 | w ; tim-UAS-Gal4 / + ; LABL / UAS-FLP2 |
| Figure 7B-7C | tim-UAS-Gal4 \| *han^5304^* | *han^5304^* ; tim-UAS-Gal4 / + ; LABL / UAS-FLP2 |
| Figure 8B-8E | elav-Gal4 | elav-Gal4 ; + / + ; LABL / UAS-FLP2 |
| Figure 8B-8E | mef2-Gal4 | w ; LABL / + ; mef2-Gal4 / UAS-FLP2 |
| Figure 8B-8E | esg-Gal4 | w ; esg-Gal4 / + ; LABL / UAS-FLP2 |
| Figure 8B-8E | NP3084-Gal4 | w ; LABL / + ; NP3084-Gal4 / UAS-FLP2 |
| Figure 8B-8E | C564-Gal4 | w ; C564-Gal4 / + ; LABL / UAS-FLP2 |
| Figure 8B-8E | LSP2-Gal4 | w ; LABL / + ; LSP2-Gal4 / UAS-FLP2 |
| Figure 8B-8E | mef2-Gal4 \| *han^5304^* | *han^5304^* ; LABL / + ; mef2-Gal4 / UAS-FLP2 |
| Figure 8B-8E | esg-Gal4 \| *han^5304^* | *han^5304^* ; esg-Gal4 / + ; LABL / UAS-FLP2 |
| Figure 8B-8E | NP3084-Gal4 \| *han^5304^* | *han^5304^* ; LABL / + ; NP3084-Gal4 / UAS-FLP2 |
| Figure 8B-8E | C564-Gal4 \| *han^5304^* | *han^5304^* ; C564-Gal4 / + ; LABL / UAS-FLP2 |
| Figure 8B-8E | LSP2-Gal4 \| *han^5304^* | *han^5304^* ; LABL / + ; LSP2-Gal4 / UAS-FLP2 |
| Figure 2-figure supplement 1 | tim-UAS-Gal4 | w ; tim-UAS-Gal4 / + ; LABL / UAS-FLP2 |
| Figure 3-figure supplement 1A-1C | iso31 | w ; + ; + |
| Figure 3-figure supplement 1A-1B | *han^5304^* | *han^5304^* ; + ; + |
| Figure 3-figure supplement 1A | *tim^01^* | w ; *tim^01^* ; + |
| Figure 4-figure supplement 1 | Clk4.1-Gal4 | w ; LABL / + ; Clk4.1-Gal4 / UAS-FLP2 |
| Figure 4-figure supplement 1 | Mai179-Gal4 | w ; Mai179-Gal4 / + ; LABL / UAS-FLP2 |
| Figure 4-figure supplement 1 | Clk9M-Gal4 | w ; Clk9M-Gal4 / + ; LABL / UAS-FLP2 |
| Figure 4-figure supplement 2 | tim-UAS-Gal4 | w ; tim-UAS-Gal4 / + ; LABL / UAS-FLP2 |
| Figure 7-figure supplement 1A | tim-UAS-Gal4 \| *per^S^* | *per^S^* ; tim-UAS-Gal4 / + ; LABL / UAS-FLP2 |
| Figure 7-figure supplement 1B | Pdf-Gal4 \| *per^S^* | *per^S^* ; Pdf-Gal4 / + ; LABL / UAS-FLP2 |
| Figure 7-figure supplement 1C | DvPdf-Gal4 \| *per^S^* | *per^S^* ; DvPdf-Gal4 / + ; LABL / UAS-FLP2 |
| Figure 7-figure supplement 1D | R18H11-Gal4 \| *per^S^* | *per^S^* ; LABL / + ; R18H11-Gal4 / UAS-FLP2 |
| Figure 7-figure supplement 1E | tim-UAS-Gal4 \| *per^L^* | *per^L^* ; tim-UAS-Gal4 / + ; LABL / UAS-FLP2 |
| Figure 7-figure supplement 1F | Pdf-Gal4 \| *per^L^* | *per^L^* ; Pdf-Gal4 / + ; LABL / UAS-FLP2 |
| Figure 7-figure supplement 1G | DvPdf-Gal4 \| *per^L^* | *per^L^* ; DvPdf-Gal4 / + ; LABL / UAS-FLP2 |
| Figure 7-figure supplement 1H | R18H11-Gal4 \| *per^L^* | *per^L^* ; LABL / + ; R18H11-Gal4 / UAS-FLP2 |
| Figure 8-figure supplement 1 | mef2-Gal4 | w ; LABL / + ; mef2-Gal4 / UAS-FLP2 |
| Figure 8-figure supplement 1 | esg-Gal4 | w ; esg-Gal4 / + ; LABL / UAS-FLP2 |
| Figure 8-figure supplement 1 | NP3084-Gal4 | w ; LABL / + ; NP3084-Gal4 / UAS-FLP2 |
| Figure 8-figure supplement 1 | C564-Gal4 | w ; C564-Gal4 / + ; LABL / UAS-FLP2 |
| Figure 8-figure supplement 1 | LSP2-Gal4 | w ; LABL / + ; LSP2-Gal4 / UAS-FLP2 |
